# Outcome with lenalidomide plus dexamethasone followed by early autologous stem cell transplantation in patients with newly diagnosed multiple myeloma on the ECOG-ACRIN E4A03 randomized clinical trial: long-term follow-up

**DOI:** 10.1038/bcj.2016.68

**Published:** 2016-09-02

**Authors:** N Biran, S Jacobus, D H Vesole, N S Callander, R Fonseca, M E Williams, R Abonour, M S Katz, S V Rajkumar, P R Greipp, D S Siegel

**Affiliations:** 1 Hackensack University Medical Center, John Theurer Cancer Center, Hackensack, NJ, USA; 2 Biostatistics and Computational Biology, Dana Farber Cancer Institute, Boston, MA, USA; 3 Hackensack University Medical Center, John Theurer Cancer Center at Hackensack UMC, Hackensack, NJ, USA; 4 University of Wisconsin Carbone Cancer Center, Madison, WI, USA; 5 Lombardi Cancer Center, Mayo Clinic, Scottsdale, AZ Georgetown University, Washington, DC, USA; 6 Division of Hematology/Oncology, University of Virginia Cancer Center, Charlottesville, VA, USA; 7 Department of Hematology/Oncology, Indiana University School of Medicine, Indianapolis, IN, USA; 8 International Myeloma Foundation, Los Angeles, CA, USA; 9 Division of Hematology, Mayo Clinic, Rochester, MN, USA; 10 Mayo Clinic, Rochester, MN, USA

## Abstract

In Eastern Cooperative Oncology Group-ACRIN E4A03, on completion of four cycles of therapy, newly diagnosed multiple myeloma patients had the option of proceeding to autologous peripheral blood stem cell transplant (ASCT) or continuing on their assigned therapy lenalidomide plus low-dose dexamethasone (Ld) or lenalidomide plus high-dose dexamethasone (LD). This landmark analysis compared the outcome of 431 patients surviving their first four cycles of therapy pursuing early ASCT to those continuing on their assigned therapy. Survival distributions were estimated using the Kaplan–Meier method and compared with log-rank test. Ninety patients (21%) opted for early ASCT. The 1-, 2-, 3-, 4- and 5-year survival probability estimates were higher for early ASCT versus no early ASCT at 99, 93, 91, 85 and 80% versus 94, 84, 75, 65 and 57%, respectively. The median overall survival (OS) in the early versus no early ASCT group was not reached (NR) versus 5.78 years. In patients <65 years of age, median OS in the early versus no early ASCT groups was NR in both, hazard ratio 0.79, 95% confidence interval: (0.50, 0.25). In patients ⩾65 years of age, median OS in the early versus no early ASCT was NR versus 5.11 years. ASCT dropped out of statistical significance (*P=*0.080). Patients opting for ASCT after induction Ld/LD had a higher survival probability and improvement in OS regardless of dexamethasone dose density.

## Introduction

High-dose chemotherapy and autologous peripheral blood stem cell transplant (ASCT) were demonstrated in large, randomized clinical trials, to improve progression-free survival (PFS) and overall survival (OS) compared with standard chemotherapy in patients with multiple myeloma (MM)^1, 2^ Other randomized trials demonstrated an improvement only in PFS with no significant OS benefit.^3, 4^ Because of the improved PFS resulting in prolonged time off treatment without symptoms with improved quality of life,^3, 5^ ASCT following induction therapy has become an integral part of treatment in patients with newly diagnosed MM (NDMM) and continues to be the recommended treatment for transplant eligible NDMM by the International Myeloma Working Group.^6^

Over the last decade, immunomodulatory agents (IMiDs) and proteasome inhibitors (PIs) have led to a significant improvement in PFS, OS and response rates for patients with NDMM.^7, 8, 9^ Because of the response rates, especially the depth of response to IMiDs and PIs, the role of ASCT in the upfront setting has become more controversial. Although most clinical practice guidelines recommend early ASCT as the standard of care in transplant eligible patients,^10, 11^ some experts suggest that standard risk patients can opt for delayed ASCT if stem cells can be cryopreserved.^12^ One prospective trial randomized 253 patients with NDMM, aged 65 years or younger, to ASCT versus melphalan, prednisone and lenalidomide consolidation after lenalidomide plus low-dose dexamethasone (Ld) induction therapy and found that consolidation with high-dose melphalan plus ASCT as compared with melphalan, prednisone and lenalidomide consolidation significantly prolonged PFS and OS.^13^ Another study that compared upfront ASCT versus chemotherapy with cyclophosphamide, dexamethasone and lenalidomide with or without maintenance showed a significant PFS advantage with upfront ASCT.^14^ However, there continues to be an increasing trend towards delaying ASCT. Hence, the timing of ASCT in the era of PIs and IMiDs is a critically important question, especially in patients over age 65 years. Preliminary results from the EMN02/H095 randomized trial of 1308 newly diagnosed MM patients show a PFS benefit of upfront ASCT versus novel agent-based that was maintained on multivariable Cox regression analysis.^15^

In the phase 3 clinical study Eastern Cooperative Oncology Group (ECOG)-ACRIN E4A03, Ld was associated with better OS and with lower toxicity compared with lenalidomide plus high-dose dexamethasone (LD) in patients with NDMM.^16^ This study demonstrated the superiority of Ld over LD and led to the widespread acceptance of Ld as a standard induction regimen. More recently, the Frontline Investigation of Revlimid and Dexamethasone versus Standard Thalidomide study has compared Ld as continuous therapy versus Ld given for 18 cycles versus melphalan plus prednisone plus thalidomide.^17^ Again, continuous Ld, as had previously been described in EOCG E4A03 and confirmed in the Frontline Investigation of Revlimid and Dexamethasone versus Standard Thalidomide trial, proved to be superior and will likely lead to a more worldwide utilization of continuous Ld as a standard induction regimen.

This analysis is the long-term follow-up to a prior *post-hoc* retrospective landmark analysis (LM) comparing outcomes of early versus no early ASCT. On completion of four cycles of therapy in the ECOG-ACRIN E4A03 study, patients had the option of proceeding with ASCT or continuing on the assigned therapy Ld or LD. In this context, the primary objective was to evaluate the outcome of the subpopulations of patients opting to pursue ASCT after four cycles of therapy relative to the population not pursuing early ASCT and either off study treatment or opting to continue with Ld or LD.

## Patients and methods

### Patients

Patients enrolled on the ECOG-ACRIN E4A03 randomized phase 3 clinical trial, the results of which have been previously published,^16^ were included in the current analysis. On E4A03, NDMM patients were randomized to LD versus Ld. Patients on both arms received oral lenalidomide 25 mg orally per day on days 1–21 of each 28-day cycle. Patients on LD received oral dexamethasone 40 mg per day on days 1–4, 9–12 and 17–20 of each 28-day cycle and patients on Ld received oral dexamethasone 40 mg per day on days 1, 8, 15 and 22 of each 28-day cycle. Median duration of therapy was 4 months in the LD group and 6 months in the Ld group, with 14% of patients in the LD group continuing on therapy >1 year versus 30% in Ld. After the first four cycles of therapy, patients could discontinue therapy, some choosing to pursue stem cell transplantation (ASCT), or continue therapy on study until disease progression. The decision for pursuing ASCT may or may not have been made after four cycles. ASCT occurred within 6 months of discontinuation of therapy for patients in the early ASCT group.

Between November 2004 and April 2006, 445 patients were enrolled on the E4A03 treatment trial from participating institutions. In the primary study,^16^ there was evidence of an OS advantage on the Ld arm compared with LD (96% versus 87%, *P<*0.001) at the first pre-planned interim analysis for response in March 2007, which occurred at a median follow-up of 12.5 months and included 108 patients. Of note, OS was not a protocol-specified endpoint. This prompted the Data Monitoring Committee to release the study data and recommend cross-over of patients. At this time, 79 patients (18%) were still on treatment and all had passed the 4-cycle primary endpoint. LD patients continuing therapy beyond 4 cycles were advised to lower the dexamethasone dose by two-thirds from 12 to 4 days per cycle. As such, longer-term OS analyses by treatment are confounded. At 3 years, the OS curves by treatment cross, yielding a nonsignificant *P*-value. The 3-year OS for both the Ld and LD arms was 75%.

### Statistical design and analysis

Per protocol, patients received a minimum of four cycles of therapy parallel to the primary endpoint of a 4-month response rate. The LM analysis that is the subject of this study included 431 patients surviving the first 4 cycles of induction therapy. For this *post*-*hoc* analysis, early ASCT was defined as ASCT occurring within 6 months of discontinuation of study-mandated therapy. Of note, patients in the ‘No Early SCT' subgroup could have received ASCT at some time. Data regarding subsequent therapies or PFS is not available.

This is a *post*-*hoc*, retrospective analysis of OS by early ASCT status overall and within age subgroups dichotomized at 65 years.

The association of early ASCT status with OS was of primary interest but additional endpoints evaluated were PFS, grade 3 or higher non-hematological toxicity and best overall response rate (⩾partial response). Survival distributions based on early ASCT status were estimated using the Kaplan–Meier method and compared with the log-rank test. PFS was defined as the time from randomization to disease progression or death due to any cause. Patients were censored at date of last disease evaluation if they did not experience an event. Cox proportional hazards regression was used to examine the relationship between baseline factors and survival outcome in univariate and adjusted models, using stepwise selection and adjusting for established prognostic factors. ASCT was evaluated as a time-varying covariate.

The response criteria used were standard European Group for Blood and Bone Marrow Transplant,^18^ except that responses were confirmed 4 weeks apart (instead of 6 weeks). Responses were classified according to the International Myeloma Working Group response criteria.^19^ As the study did not mandate bone marrow evaluation to confirm complete response, a category of immunofixation-negative complete response was defined as confirmed disappearance of the monoclonal protein in the serum and urine by immunofixation studies without the requirement for bone marrow studies. Fisher's exact test was used to compare response and toxicity rate. A *P*-value of 0.05 was considered statistically significant.

## Results

### Number and characteristics of patients included in the LM analysis

**Figure 1:**
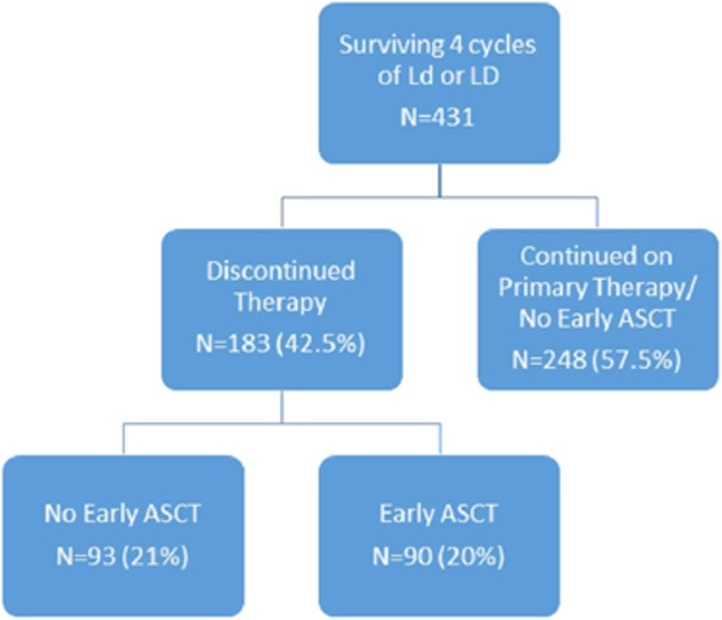
LM analysis flowchart. ASCT, autologous stem cell transplant; Ld, lenalidomide plus low-dose dexamethasone; LD, lenalidomide plus high-dose dexamethasone.

Of the 431 patients including in the LM analysis, 183 (42.5%) discontinued therapy after 4 cycles and 248 (57.5%) continued their primary therapy beyond 4 cycles (Figure 1). Of the 183 patients who discontinued primary therapy, 93 (50.8%) did not receive upfront ASCT and 90 (49%) opted for early ASCT. Of the 341 patients who did not pursue early ASCT, 93 patients went off study therapy and 248 continued primary therapy. In the group of patients who continued primary therapy, 60 eventually reported receiving ASCT. Patients in this cohort had been randomized to either Ld (*n=*179, 52.5%) or LD (*n=*162, 47.5%). Patients were separated into subgroups based on age <65 years (*n=*209, 48.5%) and age ⩾65 years (*n=*222, 51.5%) for the LM analysis. Of those who were <65 years old, 141 (67.5%) had no early ASCT and 68 (32.5%) had early ASCT. Of those who were ⩾65 years old, 200 (90%) had no early ASCT and 22 (10%) had early ASCT.

At baseline (Table 1), early ASCT patients were younger (median 57.5 vs 66 year, *P<*0.001), more fit (ECOG performance status 0, 45% vs 56%, *P=*0.096) and with less aggressive disease (ISS Stage 3, 12% vs 28%, *P=*0.002).

### OS by early ASCT status

**Figure 2:**
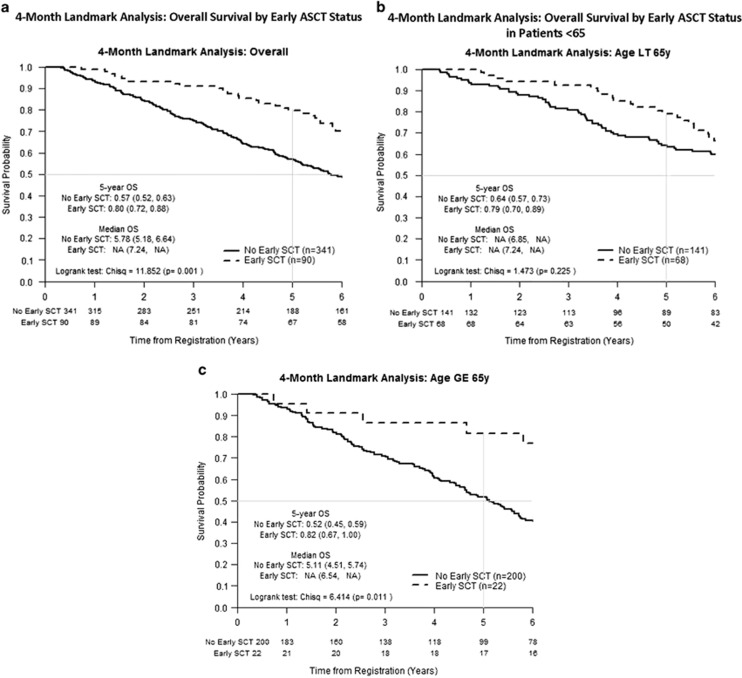
(**a**) Four-month LM analysis: OS by early ASCT status. (**b**) Four-month LM analysis: OS by early ASCT status in patients <65 years. (**c**) Four-month LM analysis: OS by early ASCT status in patients ⩾65 years.

The 1-, 2-, 3-, 4- and 5-year survival probability estimates were higher for early ASCT versus no ASCT at 99, 93, 91, 85 and 80% versus 94, 84, 75, 65 and 57%. Survival probability differences between patients in the early ASCT versus no early ASCT increased with time. Differences between survival probability at 1, 2, 3, 4 and 5 years were 5, 9, 16, 21 and 23%, respectively. The median OS in the early ASCT versus no early ASCT group was not reached (NR) versus 5.78 years, respectively (*P=*0.001; Figure 2a). Survival hazard ratio was 0.55, 95% confidence interval (95% CI): (0.38–0.80).

In the subset of patients age <65 years, the difference in survival probability between those who had early ASCT versus no early ASCT at 1, 2, 3, 4 and 5 years was 5, 6, 12, 16 and 15%, respectively. Thus, after 4 years there was no significantly continued difference in survival probability. In the subset of patients aged 65 years or older, the difference in survival probability between early ASCT and no early ASCT at 1, 2, 3, 4 and 5 years was 2, 9, 15, 25 and 30%, respectively. As such, the difference in survival probability plateaued in younger patients but increased each year in patients over 65 years.

In patients aged <65 years included in the LM analysis (*N=*209), median OS in patients who went on to early ASCT (*N=*68) versus no early ASCT (*N=*141) was NR in both (*P=*0.225; Figure 2b). Survival hazard ratio was 0.79, 95% CI: (0.50–1.25). The 5-year survival probability of those who had early ASCT versus no early ASCT was 79%, 95% CI: (70–89) versus 64%, 95% CI: (57–73).

In patients ⩾65 years of age included in the LM analysis (*N=*222), median OS in patients who had early ASCT (*N=*22) versus no early ASCT (*N=*200) was NR versus 5.11 years (*P=*0.011; Figure 2c). Survival hazard ratio was 0.42, 95% CI: (0.21–0.86). The 5-year survival probability of those who had early ASCT versus no early ASCT was 82%, 95% CI: (67–100) versus 52%, 95% CI: (45–59), respectively.

### OS by ASCT status and treatment arm

Median OS in all patients included in the LM analysis, who went on to early ASCT for those who received LD (*N=*50) versus Ld (*N=*40) was NR versus 7.24 years, respectively (*P=*0.134). For those who had no early ASCT, median OS for those who received LD (*N=*162) versus Ld (*N=*179) was 5.63 versus 5.91 years, respectively (*P=*0.131). As stated above, cross-over occurred at the first pre-planned response interim analysis and, thus, makes it impossible to make treatment comparisons.

### Multivariable analysis

As the separation of the Kaplan–Meier curves does not account for selection bias, early ASCT versus no early ASCT was evaluated in a Cox proportional hazards multivariable regression model adjusting for other known survival prognostic factors (Table 2). ASCT, which was collected for only the first 6 months post treatment, was captured as a time-varying covariate. Other variables included in the Cox proportional hazards regression model included treatment (Ld vs LD), age (<65 vs ⩾65 years), ISS Stage (I/II vs III) and ECOG performance status (0 vs 1/2). Age was the only variable to emerge as statistically significant (*P=*0.04). ASCT as a time-varying covariate fell out of significance (*P=*0.080).

### Best four-cycle response

A potentially confounding factor in assessing the outcome of patients going on to ASCT versus those who did not have an early ASCT is the level of response at the completion of four cycles of therapy. In the primary study the four-cycle overall response rate (primary endpoint) was 73.7% (311/422) and in this LM data set the rate was 74.4% (305/410). There was no statistically significant difference in the four-cycle overall response rate between patients who chose early ASCT versus no early ASCT (Table 3).

### Toxicity

Toxicity results over the four cycles of therapy could also inform outcomes. Global grade 3–4 treatment-related non-hematological toxicity is summarized in Table 4. In the primary study the 4-cycle grade 3–4 treatment-related non-hematologic toxicity rate was 39.7% (176/443) and in this LM data set the rate was 40.5% (174/430). Overall, there was a statistically significant difference in rates between early ASCT (28.9%) versus no early ASCT (43.5%), *P=*0.015. The difference widens in the older age group (18.2%) but power is limited due to small sample size.

## Discussion

The ECOG-ACRIN E4A03^16^ demonstrated superior survival probabilities for patients undergoing early ASCT relative to those who did not have early ASCT (within 6 months of completing of the fourth cycle of therapy). This was true at 1, 2, 3, 4 and 5 years post study entry. The difference in survival probabilities between early ASCT and no early ASCT increased with each year of follow-up, with a doubling of the difference in the subset of patients aged 65 years or older between years 3 and 5. However, caution should be used in the interpretation of the subgroup analyses given the lack of randomization, the lack of data on salvage therapies, physician and patient bias, and the *post-hoc* nature of this analysis. As an example, the cohort of patients aged ⩾65 years, those in the early ASCT group compared with the no early ASCT group were younger, more fit (less ECOG performance status 1–2) and had less aggressive disease (less ISS stage 3). In multivariable regression analysis attempting to correct for established prognostic factors, ASCT as a time-varying covariate only showed a trend toward improvement (*P=*0.08).

The choice of early ASCT appeared to attenuate much of the increased risk of death that occurred in patients assigned to the LD arm of the trial. One potential explanation for the superiority of outcome in those undergoing early ASCT is that those who went on to early ASCT were destined to have a more favorable prognosis due to factors such as age, baseline performance status, improved response after four cycles of therapy (time point at which treatment decision was made) or lower treatment-related toxicity over the first four cycles of therapy. This LM analysis attempts to address these explanations, which would favorably bias the early ASCT group.

By choosing this four-cycle LM at which to start the analysis, we attempted to decrease the bias introduced by early deaths being assigned to the non-ASCT arm. The LM analysis continued to demonstrate that patients who went on to early ASCT after induction lenalidomide and dexamethasone had a lower 1, 2, 3, 4 and 5-year mortality rate and corresponding improvement in OS regardless of dexamethasone dose density (Ld or LD). This study is subject to the usual limitations of a non-randomized study. The relatively small numbers in overall and within each subset of age cohort may have prevented the differences in survival to have reached statistical significance and may have been the reason for ASCT to have fallen out of significance in multivariable analysis.

The overwhelming majority of deaths related to progressive MM occur beyond 1 year from the initiation of therapy. Thus, 1-year mortality was used as a surrogate for treatment-related mortality. The presumption that treatment-related mortality should be greater for older patients undergoing ASCT is addressed by looking at the age >65 years cohorts, where 1-year mortality is similar between the early ASCT population and the no early ASCT population. Although the number of older patients was not large, there is no suggestion that older patients cannot do as well as younger patients with proper patient selection. Transplant can be effective and safely conducted in an older patient population and the notion that older patients are not candidates for ASCT is not supported by this limited data set.

In patients <65 years of age undergoing early ASCT, the increased early risk of death associated with LD appears to be dampened. This must be interpreted with caution, as it is possible that patients who experienced significant toxicity with LD were unable to go on to ASCT and therefore were included in the no early ASCT group.

Novel therapies have brought into question the role and, more specifically, the timing of ASCT. Two older randomized studies compared outcomes with early versus delayed transplant, both completed before the incorporation of IMiD- or PI-based agents into treatment algorithms. Fermand *et al.*^3^ showed an improvement in event-free survival and improvement in quality of life in terms of time without symptoms, treatment or treatment toxicity in patients transplanted early, although there was no OS benefit. Similarly, Barlogie *et al.*^20^ compared high-dose therapy with melphalan 140 mg/m^2^ and total body irradiation 12 cGy to maintenance with vincristine, carmustine, melphalan, cyclophosphamide and prednisone. On disease progression, the patients in the vincristine, carmustine, melphalan, cyclophosphamide and prednisone arm were to receive ASCT. There was no difference in response rate, PFS or OS between arms, possibly due to an inferior transplant preparative regimen. One trial has evaluated the role of ASCT versus no transplant with the use of lenalidomide as part of therapy. After induction with RD for 4 cycles, 273 patients <65 years of age with newly diagnosed MM were randomized to either consolidation of melphalan, prednisone and lenalidomide for 6 cycles or to tandem ASCT.^13^ ASCT was to be performed in the non-transplant arm at time of disease progression. With a median follow-up of 51.2 months, both PFS and 4-year OS were significantly longer in the ASCT arm. Of those randomized to melphalan, prednisone and lenalidomide, only 63% received the planned ASCT at first relapse, which may have led to the significant difference in OS. Gay *et al.*^14^ randomized 389 newly diagnosed transplant-eligible MM patients to ASCT versus cyclophosphamide-dex-lenalidomide chemotherapy after induction. PFS during consolidation was significantly shorter with chemotherapy plus lenalidomide compared with ASCT (median 28.6 versus 43.3 months, *P<*0.0001).^14^

Prospective, randomized clinical trials evaluating the outcomes of early versus delayed ASCT in the era of IMiD and PI-based therapies are ongoing (IFM-DFCI 2009 study NCT01208662 and the European Intergroup Trial). The IFM/DFCI 2009/CTN 1304 parallel phase 3 study randomized newly diagnosed MM patients to induction therapy with bortezomib, lenalidomide and dexamethasone (VRD) × 3 cycles and stem cell mobiliziation followed by either ASCT and two cycles of RVD consolidation or five cycles of RVD consolidation with lenalidomide maintenance for 1 year in both arms. Results from a pre-specified interim analysis with a median follow-up of 39 months showed an improvement in 3-year PFS with ASCT versus RVD (61% versus 48%, respectively, *P<*0.0002) and was uniform across all subgroups.^21^ The 3-year post-randomization OS rate was extremely high (88%) and similar between the two study groups. Further results of this trial and other large prospective studies are needed to assess the role and timing of ASCT.

In conclusion, the ECOG-ACRIN E4A03 demonstrated superior survival probabilities for patients undergoing early ASCT relative to those who did not have early ASCT. This was true in patients of all age subgroups and maintained significance in patients ⩾65 years, although in multivariable analysis adjusting for the differences in cohorts as a result of subgroup analysis ASCT fell out of significance. The notion that the superior outcome for patients undergoing early ASCT is due to the differences in age between the two populations is not supported by this data.

## Figures and Tables

**Table 1:** Baseline characteristics of patients included in the LM analysis for OS

*Cohort*		*Age<65 years*		*Age* ⩾ *65 years*		*Total*		
*Variable*		*No early SCT*	*Early SCT*	*No early SCT*	*Early SCT*	*No early SCT*	*Early SCT*	P*-value*
Total		141	68	200	22	341	90	
Age (year)	Median (Q1,Q3)	57 (53,61)	54 (49,59)	72 (68,76)	67 (66,70)	66 (59,73)	58 (51,64)	<0.001
Gender: male	*N* (%)	79 (56.0)	36 (52.9)	110 (55.0)	16 (72.7)	189 (55.4)	52 (57.8)	0.721
Race: White	*N* (%)	114 (80.9)	60 (88.2)	177 (88.5)	18 (81.8)	291 (85.3)	78 (86.7)	0.866
ISS: stage III	*N* (%)	26 (19.3)	6 (9.5)	65 (34.8)	4 (19.1)	91 (28.3)	10 (11.9)	0.002
Unk		6	5	13	1	19	6	
ECOG PS: 1–2	*N* (%)	75 (53.2)	29 (42.6)	112 (56.0)	11 (50.0)	187 (54.8)	40 (44.4)	0.096
Bone disease: present	*N* (%)	89 (63.1)	49 (72.1)	112 (56.0)	16 (72.7)	201 (58.9)	65 (72.2)	0.021
FISH: high risk	*N* (%)	7 (15.9)	4 (15.4)	8 (18.2)	2 (22.2)	15 (17.0)	6 (17.1)	1.000
Unk		97	42	156	13	253	55	
β-2 Microglobulin (μg/ml)	Median (Q1,Q3)	3.4 (2.4,4.6)	2.8 (2.3,3.8)	4.3 (2.7,6.7)	4.1 (3.5,5.2)	3.8 (2.6,5.7)	3.1 (2.4,4.3)	0.004
Unk		4	1	3	0	7	1	
Hemoglobin (g/dL)	Median (Q1,Q3)	10.9 (9.7,12.3)	11.8 (9.9,12.7)	10.9 (9.6,11.9)	11.3 (9.9,12.6)	10.9 (9.6,12.1)	11.7 (9.9,12.7)	0.017

Abbreviations: ECOG PS, Eastern Cooperative Oncology Group performance status; FISH, fluorescent *in situ* hybridization; LM, landmark; OS, overall survival; SCT, stem cell transplantation.

**Table 2:** Cox proportional hazards model for survival: multivariable

		*HR (95% CI)*	P*-value*
Adjusted	Ld vs LD	1.06 (0.81–1.39)	0.673
	SCT tvc^a^	0.71 (048–1.04)	0.080
	Age (<65 vs ⩾65 years)	0.66 (0.49–0.88)	**0.004**
	ISS stage (I/II vs III)	0.67 (0.50–0.91)	0.010
	ECOG PS (0 vs 1/2)	0.74 (0.56–0.97)	0.031

Abbreviations: ASCT, autologous peripheral blood stem cell transplant; CI, confidence interval; ECOG PS, Eastern Cooperative Oncology Group performance status; HR, hazard ratio; Ld, low-dose dexamethasone; LD, high-dose dexamethasone; SCT, stem cell transplantation; tvc, time-varying covariate.

a ASCT and tvc. Bold value indicates statistical significance.

**Table 3:** Best response ⩾PR at 4 months (primary endpoint)

	*No early SCT% (freq/n)*	*Early SCT% (freq/n)*	*Absolute difference*	*Odds ratio (95% CI)*	*Fisher's exact* P*-value*
Overall	75.4% (242/321)	70.8% (63/89)	4.6%	1.26 (0.75, 2.13)	0.411
Age<65 years	75.7% (103/136)	70.6% (48/68)	5.1%	1.30 (0.68, 2.50)	0.499
Age ⩾ 65 years	75.1% (139/185)	71.4% (15/21)	3.7%	1.21 (0.44, 3.30)	0.791

Abbreviations: CI, confidence interval; PR, partial remission; SCT, stem cell transplant.

**Table 4:** Global grade 3–4 treatment-related non-hematological toxicity

	*No early SCT % (freq/n)*	*Early SCT % (freq/n)*	*Absolute difference*	*Fisher's exact* P*-value*
Overall	43.5% (148/340)	28.9% (26/90)	14.6%	0.015
Age<65 years	34.0% (48/141)	27.9% (19/68)	6.1%	0.431
Age ⩾65 years	50.0% (100/200)	31.8% (7/22)	18.2%	0.120

Abbreviations: CI, confidence interval; SCT, stem cell transplant.

## References

[bib1] Child JA, Morgan GJ, Davies FE, Owen RG, Bell SE, Hawkins K et al. High-dose chemotherapy with hematopoietic stem-cell rescue for multiple myeloma. N Engl J Med 2003; 348: 1875–1883. 12736280 10.1056/NEJMoa022340

[bib2] Attal M, Harousseau JL, Stoppa AM, Sotto JJ, Fuzibet JG, Rossi JF et al. A prospective, randomized trial of autologous bone marrow transplantation and chemotherapy in multiple myeloma. Intergroupe Francais du Myelome. N Engl J Med 1996; 335: 91–97. 8649495 10.1056/NEJM199607113350204

[bib3] Fermand JP, Katsahian S, Divine M, Leblond V, Dreyfus F, Macro M et al. High-dose therapy and autologous blood stem-cell transplantation compared with conventional treatment in myeloma patients aged 55 to 65 years: long-term results of a randomized control trial from the Group Myelome-Autogreffe. J Clin Oncol 2005; 23: 9227–9233. 16275936 10.1200/JCO.2005.03.0551

[bib4] Blade J, Rosinol L, Sureda A, Ribera JM, Diaz-Mediavilla J, Garcia-Larana J et al. High-dose therapy intensification compared with continued standard chemotherapy in multiple myeloma patients responding to the initial chemotherapy: long-term results from a prospective randomized trial from the Spanish cooperative group PETHEMA. Blood 2005; 106: 3755–3759. 16105975 10.1182/blood-2005-03-1301

[bib5] Fermand JP, Ravaud P, Chevret S, Divine M, Leblond V, Belanger C et al. High-dose therapy and autologous peripheral blood stem cell transplantation in multiple myeloma: up-front or rescue treatment? Results of a multicenter sequential randomized clinical trial. Blood 1998; 92: 3131–3136. 9787148

[bib6] Ludwig H, Miguel JS, Dimopoulos MA, Palumbo A, Garcia Sanz R, Powles R et al. International Myeloma Working Group recommendations for global myeloma care. Leukemia 2014; 28: 981–992. 24177258 10.1038/leu.2013.293

[bib7] Kumar SK, Rajkumar SV, Dispenzieri A, Lacy MQ, Hayman SR, Buadi FK et al. Improved survival in multiple myeloma and the impact of novel therapies. Blood 2008; 111: 2516–2520. 17975015 10.1182/blood-2007-10-116129 PMC2254544

[bib8] Jagannath S, Richardson PG, Barlogie B, Berenson JR, Singhal S, Irwin D et al. Bortezomib in combination with dexamethasone for the treatment of patients with relapsed and/or refractory multiple myeloma with less than optimal response to bortezomib alone. Haematologica 2006; 91: 929–934. 16818280

[bib9] Rajkumar SV, Hayman SR, Lacy MQ, Dispenzieri A, Geyer SM, Kabat B et al. Combination therapy with lenalidomide plus dexamethasone (Rev/Dex) for newly diagnosed myeloma. Blood 2005; 106: 4050–4053. 16118317 10.1182/blood-2005-07-2817 PMC1895238

[bib10] Anderson KC, Alsina M, Bensinger W, Biermann JS, Chanan-Khan A, Cohen AD et al. NCCN clinical practice guidelines in oncology: multiple myeloma. J Natl Compr Canc Netw 2009; 7: 908–942. 19878637 10.6004/jnccn.2009.0061

[bib11] Moreau P, San Miguel J, Ludwig H, Schouten H, Mohty M, Dimopoulos M et al. Multiple myeloma: ESMO Clinical Practice Guidelines for diagnosis, treatment and follow-up. Ann Oncol 2013; 24(Suppl 6): vi133–vi137. 23956208 10.1093/annonc/mdt297

[bib12] Vincent Rajkumar S. Multiple myeloma: 2014 Update on diagnosis, risk-stratification, and management. Am J Hematol 2014; 89: 999–1009. 25223428 10.1002/ajh.23810

[bib13] Palumbo A, Cavallo F, Gay F, Di Raimondo F, Ben Yehuda D, Petrucci MT et al. Autologous transplantation and maintenance therapy in multiple myeloma. N Engl J Med 2014; 371: 895–905. 25184862 10.1056/NEJMoa1402888

[bib14] Gay F, Oliva S, Petrucci MT, Conticello C, Catalano L, Corradini P et al. Chemotherapy plus lenalidomide versus autologous transplantation, followed by lenalidomide plus prednisone versus lenalidomide maintenance, in patients with multiple myeloma: a randomised, multicentre, phase 3 trial. Lancet Oncol 2015; 16: 1617–1629. 26596670 10.1016/S1470-2045(15)00389-7

[bib15] Cavo M, Palumbo A, Zweegman S, Dimopoulos MA, Hajek R, Pantani L et al. Upfront autologous stem cell transplantation (ASCT) versus novel agent-based therapy for multiple myeloma (MM): a randomized phase 3 study of the European Myeloma Network (EMN02/H095 MM Trial). J Clin Oncol 2016; 34(suppl; abstr 8000).

[bib16] Rajkumar SV, Jacobus S, Callander NS, Fonseca R, Vesole DH, Williams ME et al. Lenalidomide plus high-dose dexamethasone versus lenalidomide plus low-dose dexamethasone as initial therapy for newly diagnosed multiple myeloma: an open-label randomised controlled trial. Lancet Oncol 2010; 11: 29–37. 19853510 10.1016/S1470-2045(09)70284-0 PMC3042271

[bib17] Benboubker L, Dimopoulos MA, Dispenzieri A, Catalano J, Belch AR, Cavo M et al. Lenalidomide and dexamethasone in transplant-ineligible patients with myeloma. N Engl J Med 2014; 371: 906–917. 25184863 10.1056/NEJMoa1402551

[bib18] Blade J, Samson D, Reece D, Apperley J, Bjorkstrand B, Gahrton G et al. Criteria for evaluating disease response and progression in patients with multiple myeloma treated by high-dose therapy and haemopoietic stem cell transplantation. Myeloma Subcommittee of the EBMT. European Group for Blood and Marrow Transplant. Br J Haematol 1998; 102: 1115–1123. 9753033 10.1046/j.1365-2141.1998.00930.x

[bib19] Durie BG, Harousseau JL, Miguel JS, Blade J, Barlogie B, Anderson K et al. International uniform response criteria for multiple myeloma. Leukemia 2006; 20: 1467–1473. 16855634 10.1038/sj.leu.2404284

[bib20] Barlogie B, Kyle RA, Anderson KC, Greipp PR, Lazarus HM, Hurd DD et al. Standard chemotherapy compared with high-dose chemoradiotherapy for multiple myeloma: final results of phase III US Intergroup Trial S9321. J Clin Oncol 2006; 24: 929–936. 16432076 10.1200/JCO.2005.04.5807

[bib21] Attal ML-CV, Hulin C, Facon T, Caillot D, Escoffre M, Arnulf B et al. Autologous Transplantation for Multiple Myeloma in the Era of New Drugs: A Phase III Study of the Intergroupe Francophone Du Myelome (IFM/DFCI 2009 Trial). American Society of Hematology: Orlando, FL, USA, 2015.

